# Gender Differences in Clinicoepidemiological Features of Vitiligo: A Cross-Sectional Analysis

**DOI:** 10.1155/2014/186197

**Published:** 2014-02-13

**Authors:** Sharmila Patil, Manjyot Gautam, Nitin Nadkarni, Neha Saboo, Kiran Godse, Maninder Singh Setia

**Affiliations:** ^1^Department of Dermatology, Dr. D. Y. Patil Medical College, Nerul, Navi Mumbai, India; ^2^Consultant Dermatologist and Epidemiologist, Mumbai, India

## Abstract

*Background*. Vitiligo has important clinical and social consequences particularly in the pigmented skin. The present study was conducted to assess the differences in clinicoepidemiological presentation of vitiligo in males and females and to understand the factors associated with spread of vitiligo in them. *Methods*. This is a cross-sectional analysis of secondary clinical data of 168 vitiligo patients at a tertiary medical centre at Navi Mumbai. We used logistic regression models to estimate the association between gender and clinical characteristics of vitiligo and to evaluate the factors associated with spread of vitiligo. *Results*. There were no significant differences between the mean ages of males and females; however, males reported a longer duration of disease (6.9 (10.4) years) compared with females (4.9 (7.4) years). Males were significantly more likely to report a family history of vitiligo compared with females (adjusted OR (aOR): 16.87, 95% CI: 2.16 to 131.69). Even though females were more likely to report spread of lesions, the association was not statistically significant (OR: 1.21, 95% CI: 0.62 to 2.36). *Discussion*. The differences in the clinical presentations between genders highlight the need to understand the different factors (possibly genetic) that may play a part in the pathogenesis of this multifactorial disease in males and females.

## 1. Introduction

Vitiligo, a common dermatological disorder is characterized by milky-white depigmented macules devoid of identifiable melanocytes. Its incidence varies from 1 to 2% worldwide [1] and has been shown to be as high as 3-4% in India [2]. Vitiligo, often considered as a multifactorial disease [3], has important clinical and social consequences particularly in the pigmented skin. Though, the condition is cosmetically important, studies have also shown the association of vitiligo with several organ specific as well as systemic autoimmune diseases [4–6].

Numerous Indian studies have highlighted the clinical profile of vitiligo in various clinical settings [7–11]. Indeed, it has been reported that the mean duration of the disease in most of the patients was less than five years and vitiligo vulgaris was the most common clinical presentation [8, 10, 12]. Epidemiological studies have given a conflicting view of the occurrence of the condition in both genders. For instance, some studies have found that vitiligo was more common in males whereas others have found it to be more common in females [5, 8–10, 12–16]. And finally, some have found no difference in both genders [1, 2, 4]. Furthermore, studies have highlighted the stigma associated with vitiligo—particularly in women [17, 18].

Though, as stated above, studies have discussed few clinical and social aspects of vitiligo in men and women, few studies have differentiated the clinicepidemiological profile between both sexes. An understanding of the condition in both sexes will help us assess the similarities and differences of the clinical condition; this knowledge may help us devise strategies for clinical care of patients. Another issue that may help us in therapeutic decisions is the knowledge of factors associated with the spread of the disease in these patients.

Thus, with the above background, we conducted the following study to: (1) assess the differences in clinicoepidemiological presentation of vitiligo in males and females and (2) to understand the factors associated with the spread of vitiligo in both males and females.

## 2. Methods

The present study is a cross-sectional analysis of secondary clinical data of 168 vitiligo patients over a period of two years (2007 to 2009).

### 2.1. Study Site

The study was conducted at the D. Y. Patil Medical College and Research Centre at Navi Mumbai, India. It is a tertiary care medical centre situated about 30 kilometres from Mumbai. The Dermatology Department of this Institute handles about 100 patients in the Outpatient Department daily and has various specialty clinics including the pigmentation and vitiligo clinic. Clinical information was recorded in all patients diagnosed with vitiligo. This included the baseline history, investigations, treatment given, and follow-up history of the patients.

### 2.2. Variables

Clinical baseline data of patients diagnosed with vitiligo were analysed for the present study. The parameters included for the analyses were demographic characteristics (age, sex, and occupation); age of onset of vitiligo and site of the initial lesion; clinical characteristics (type of vitiligo, site of the lesion, spread, and pigmentation); family history; and treatment history.

### 2.3. Data Analysis

Data were entered in MsExcel (Microsoft, Seattle, USA) and converted to Stata Version 11 (StataCorp, College Station, TX, USA) for further analysis.

We calculated proportions for categorical variables and means and standard deviations (SD) for linear variables. The differences in the proportions were assessed using the chi-square test and differences in the means were assessed using the *t*-test. Two main types of analysis were done to answer our research questions: (1) characteristics of vitiligo according to gender and (2) factors associated with spread of vitiligo.

We calculated the proportions of individuals according to their vitiligo characteristics separately for both genders; these proportions were compared using the chi-square test. We then used logistic regression models to assess the association between gender and vitiligo characteristics. The models were built in the following order: (1) unifactorial models and (2) age adjusted models. We also used logistic regression models to evaluate the factors associated with spread of vitiligo in these patients. These models were also built in the following sequence: (1) unifactorial models and (2) age and sex adjusted models.

The present analysis was approved by the Ethics Committee of our Institute. (Dr. D. Y. Patil Medical College Ethics Committee) for secondary data analysis.

## 3. Results

**Figure 1:**
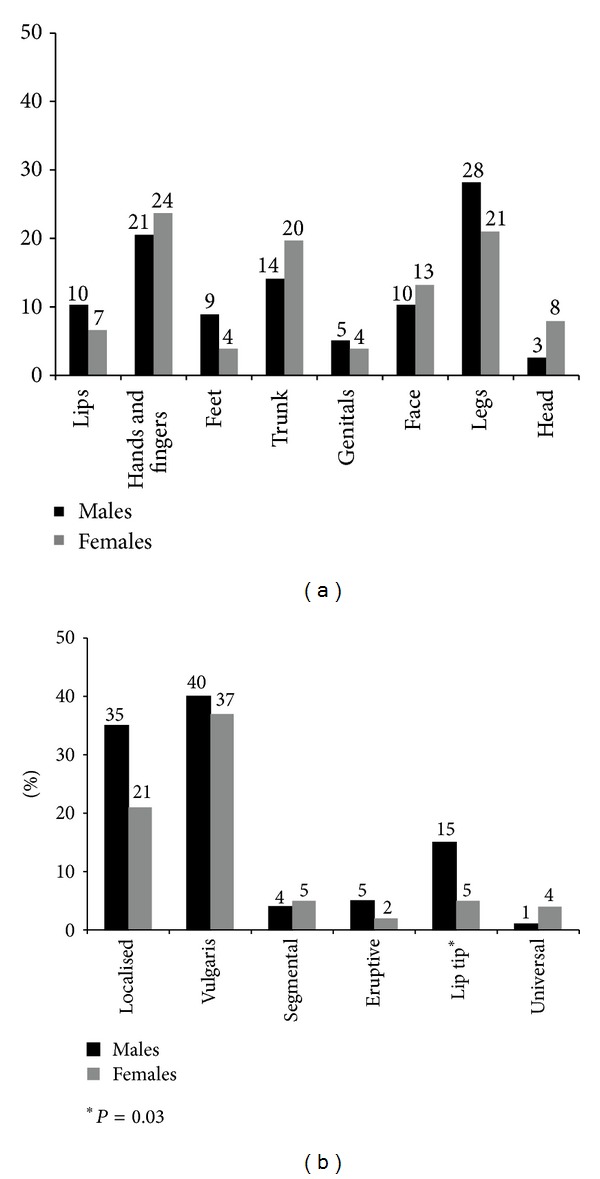
Figures showing (a) the site of initial lesion and (b) clinical presentation of vitiligo in 84 male and 84 female patients, Navi Mumbai.

There were no significant differences between the mean ages (SD) of the male patients (28.4 (15.5) years) and female patients (30.0 (14.8) years). Most of the male patients were students (35%), whereas a majority of females were housewives (64%). We found that the mean age (SD) of onset was later in females (24.8 (15.3) years) compared with males (21.2 (16.6) years); this difference was, however, not statistically significant (*P* = 0.15). Males also reported a longer duration of disease (6.9 (10.4) years) compared with females (4.9 (7.4) years). There were no significant differences in site of initial lesion in male and females (Figure 1(a)). However, males were significantly more likely to have a clinical diagnosis of lip-tip vitiligo compared with females (23% versus 2%, *P* < 0.001) (Figure 1(b)). We have described certain selected demographics and clinical characteristics in males and female patients in Table 1 and Figures 1(a) and 1(b).

We found that older males were less likely to report with vitiligo in our clinic compared with older females. Males were significantly less likely to report onset of vitiligo in the age of 25 to 45 years compared with females in our population (odds ratio (OR): 0.38, 95% confidence intervals (CI): 0.19 to 0.79). We also found that males were significantly more likely to report a family history of vitiligo compared with females (adjusted OR (aOR): 16.87, 95% CI: 2.16, 131.69). Males were also significantly more likely to have depigmented lesions (aOR: 1.99, 95% CI: 1.07 to 3.71) and leucotrichia (aOR: 8.72, 95% CI: 1.06 to 71.53) compared with females. We also found that Koebner's phenomenon was more likely to be seen in male vitiligo patients compared with female patients (aOR: 12.14, 95% CI: 2.73 to 54.09). We have described the unadjusted and adjusted association between sex and vitiligo characteristics in Table 2.

In our analysis, we found that patients who had presented to clinic after 45 years of age were less likely to report spread of lesions compared with patients who aged 12 years or less (OR: 0.51, 95% CI: 0.14 to 1.83). Even though females were more likely to report spread of lesions, the association was not statistically significant (OR: 1.21, 95% CI: 0.62 to 2.36). We found that the spread of vitiligo patches was more likely in patients who reported that the initial site of their lesions was on extremities (hands and fingers, legs, and feet) compared with those in whom the initial site of lesion was “head.” Even after adjusting for age and sex, we found that patients who had a clinical diagnosis of vitiligo vulgaris were more likely to have spread of lesions compared with those who did not have this diagnosis (aOR: 2.44, 95% CI: 1.16 to 5.17). We have described certain characteristics associated with the spread of vitiligo lesions (unadjusted and adjusted association between vitiligo and age and sex) in these patients in Table 3.

## 4. Discussion

The present cross-sectional analysis of 168 vitiligo patients provides important gender differences. Our sample of vitiligo patients had an equal proportion of males and females. Even though female patients were older compared with males, the latter reported a longer duration of disease. Males were significantly more likely to report a family history of vitiligo compared with females. They were also more likely to have a presentation with depigmented lesions, leucotrichia, and Koebner's phenomenon. The lesions which started on the extremities and those clinically diagnosed as *vitiligo vulgaris* were more likely to spread in our patients.

The clinical presentation of vitiligo had some similarities and some differences in both genders. For instance, the proportion of men and women presenting with vitiligo was similar in our sample. However, we did find that age of onset was slightly later in females compared with males; consequently, males also reported a longer duration of disease compared with females in our population. Women are more likely to report early to clinic due to cosmetic and social reasons; thus, they may have a shorter duration of disease compared with males. Compared with our study, others have reported a later age of onset of vitiligo [7, 19]. Indeed, Howits and colleagues found the age of onset to be between 40 and 60 years [19]. The proportion of cases in both genders has not shown any consistent pattern in the literature. While some authors have found a higher proportion in males, others have reported a higher proportion in females, and finally others have found no difference in the proportion between both genders [1, 2, 4, 5, 8–10, 12–16].

One important difference, however, between both genders was the type of vitiligo and a history of the condition in the family members. Indeed, we did find that males were more likely to report “hard-to-treat" type of vitiligo. They were more likely to have lip-tip type of vitiligo, leucotrichia, and depigmented lesions. Thus, males were more likely to have lesions with poor therapeutic prognosis compared with females. One possible explanation for this feature could be that patients with treatment resistant vitiligo often present to tertiary care centres like our hospital. In such a situation, however, there should be no gender difference in presentation of treatment resistant cases. Thus, potentially, a higher proportion of men have treatment resistant cases. The proportion of patients who reported a family history of vitiligo was similar to the proportion reported in the literature [15, 20]. An important finding, however, was the significantly higher proportion of men reporting a family history of vitiligo. Interestingly, Surekha and colleagues have also hypothesised that vitiligo may be X-linked [16]. Thus, our clinicepidemiological findings may have a biological basis and should be explored in detail in the Indian population.

In our population, a majority reported that the lesions started on the extremities, a finding also reported by others authors [13, 21]. One explanation could be that these sites, being trauma prone, may develop vitiligo lesions more easily in genetically predisposed persons as compared with other sites. Furthermore, lesions which started on the extremities were more likely to spread. Incidentally, we did not find any significant association between Koebner's phenomenon and spread of lesions. Thus, it is likely that the higher spread of lesions on the extremities may not be entirely explained by Koebnerization but may be due to the pathogenesis of the disease itself.

The study was not without its limitations. It was clinic based study; thus, some of the findings may not be representative of the general population. A population based sample will be useful to understand the exact prevalence of the condition and clinical features in the population at large. Since it was a secondary data analysis of data collected at the clinic, we did not have control over the variables and clinical information that we could analyse in the present manuscript. For instance, even though we had information on the history of similar lesions in the family members, we did not have further information about these members—as in were they siblings, male relatives, female relative, and so forth. Also, the family members were not examined by us. Thus, potentially, this is an important area for further exploration.

In spite of the above limitations, the study is a useful contribution to the clinicoepidemiological literature on vitiligo. We have stratified the analyses by gender to understand the similarities or differences between males and females. The differences in the clinical presentations between genders highlight the need to understand the different factors (possibly genetic) that may play a part in the pathogenesis of this multifactorial disease in males and females.

## Figures and Tables

**Table 1:** Table showing the demographics and clinical characteristics of vitiligo in male and female patients, Navi Mumbai, India.

Characteristics	All	Males	Females	
	*n* (%)	*n* (%)	*n* (%)	
	84 (100)	84 (100)	168 (100)	
Age (yrs)				
Mean (SD)	29.2 (15.2)	28.4 (15.5)	30.0 (14.8)	0.49
Occupation				
Student	46 (31)	27 (35)	19 (26)	
Housewife	48 (32)	0 (0)	47 (64)	
Labour	13 (9)	12 (16)	1 (1)	
Service	25 (17)	20 (26)	6 (8)	
Others	18 (12)	18 (23)	0 (0)	<0.001
Age of onset (yrs)				
Mean (SD)	22.9 (15.9)	21.2 (16.6)	24.8 (15.3)	0.15
Duration of disease (yrs)				
Mean (SD)	5.9 (9.0)	6.9 (10.4)	4.9 (7.4)	0.17
Lesion spreading				
Yes	118 (71)	57 (69)	61 (73)	
No	49 (29)	26 (31)	23 (27)	0.11
Family history of vitiligo				
Yes	15 (9)	14 (17)	1 (1)	
No	151 (91)	68 (83)	83 (99)	<0.001
Treatment taken for vitiligo				
Yes	82 (49)	41 (49)	41 (49)	
No	85 (51)	42 (51)	43 (51)	0.93
Trichrome				
Yes	19 (11)	10 (12)	9 (11)	
No	148 (89)	73 (88)	75 (89)	0.79
Pentachrome				
Yes	2 (1)	2 (2)	0 (0)	
No	165 (99)	81 (98)	84 (100)	0.15
Depigmented				
Yes	76 (46)	45 (54)	31 (37)	
No	91 (55)	38 (46)	53 (63)	0.03
Hypopigmented				
Yes	8 (5)	3 (4)	5 (6)	
No	159 (95)	80 (96)	79 (94)	0.48
Previtiligo				
Yes	2 (1)	1 (1)	1 (1)	
No	165 (99)	82 (99)	83 (99)	0.99
Leucotrichia				
Yes	9 (5)	8 (10)	1 (1)	
No	158 (95)	75 (90)	87 (99)	0.02
Follicular repigmentation				
Yes	5 (3)	5 (6)	0 (0)	
No	162 (97)	78 (94)	84 (100)	0.02
Koebner's phenomenon				
Yes	21 (13)	19 (23)	2 (2)	
No	146 (87)	64 (77)	82 (98)	<0.001

**Table 2:** Table showing the association between sex and characteristics of vitiligo in 168 patients in Navi Mumbai, India*.

Characteristics	Unadjusted models	Adjusted models**
Age (years)		
0–12	1.00 (0.43–2.31)	
12.1–25	1.71 (0.86–3.39)	
25.1–45	0.82 (0.44–1.52)	
>45	0.62 (0.26–1.48)	
Age of onset (years)		
0–12	1.81 (0.91–3.57)	
12.1–25	1.19 (0.61–2.38)	
25.1–45	0.38 (0.19–0.79)	
>45	1.32 (0.47–3.74)	
Site of initial lesion		
Head	0.31 (0.06–1.57)	0.32 (0.06–1.63)
Lips	1.62 (0.51–5.20)	1.64 (0.51–5.26)
Hands and fingers	0.83 (0.39–1.78)	0.86 (0.40–1.88)
Feet	2.40 (0.60–9.65)	2.17 (0.53–8.93)
Trunk	0.67 (0.28–1.56)	0.67 (0.29–1.58)
Genitals	1.32 (0.28–6.08)	1.29 (0.28–6.01)
Face	0.75 (0.28–2.03)	0.72 (0.27–1.96)
Legs	1.47 (0.70–3.09)	1.46 (0.69–3.06)
Type of vitiligo		
Localised	1.97 (0.94–4.15)	1.97 (0.94–4.17)
Vulgaris	1.12 (0.60–2.11)	1.14 (0.61–2.13)
Segmental	0.75 (0.16–3.46)	0.71 (0.15–3.29)
Eruptive	2.08 (0.37–11.65)	2.14 (0.38–12.07)
Lip-tip	3.38 (1.04–10.96)	3.47 (1.07–11.29)
Universal	0.33 (0.03–3.23)	0.34 (0.03–3.32)
Family history of vitiligo	17.09 (2.19–>100)	16.87 (2.16–131.69)
H/o spread	0.82 (0.42–1.61)	0.83 (0.43–1.62)
Trichrome	1.14 (0.44–2.97)	1.11 (0.42–2.90)
Depigmented	2.02 (1.09–3.76)	1.99 (1.07–3.71)
Hypopigmented	0.59 (0.14–2.56)	0.59 (0.14–2.55)
Previtiligo	1.01 (0.06–16.46)	0.97 (0.06–15.87)
Leucotrichia	8.85 (1.08–72.46)	8.72 (1.06–71.53)
Koebner's phenomenon	12.17 (2.73–54.18)	12.14 (2.73–54.09)

*The reference for each model is sex: female. Thus, for family h/o vitiligo, the adjusted odds ratio is 16.87 (95% confidence intervals 2.16 to 131.69) compared with females.

**Adjusted for age.

**Table 3:** Table showing the association between vitiligo characteristics and spread of vitiligo in 168 patients, Navi Mumbai, India.

Characteristics	Unadjusted models	Adjusted models*
Age (years)		
0–12	Reference	
12.1–25	0.38 (0.12–1.20)	
25.1–45	0.67 (0.22–2.05)	
>45	0.51 (0.14–1.83)	
Sex		
Males	Reference	
Females	1.21 (0.62–2.36)	
Age of onset		
0–12	Reference	
12.1–25	1.01 (0.42–2.41)	
25.1–45	1.32 (0.53–3.29)	
>45	1.29 (0.36–4.64)	
Site of initial lesion		
Head	Reference	Reference
Lips	3.75 (0.59–23.94)	4.53 (0.68–30.27)
Hands and fingers	4.62 (0.91–23.43)	5.11 (0.99–26.43)
Feet	15.0 (1.21–>100)	20.88 (1.56–>100)
Trunk	3.75 (0.72–19.64)	4.21 (0.79–22.59)
Genitals	0.28 (0.02–3.58)	0.32 (0.02–4.25)
Face	4.33 (0.74–25.29)	5.10 (0.84–30.88)
Legs	7.38 (1.41–38.42)	8.96 (1.64–49.02)
Family history of vitiligo		
No	Reference	Reference
Yes	1.75 (0.47–6.51)	2.04 (0.53–7.90)
Type of vitiligo		
Localised	0.50 (0.23–1.10)	0.50 (0.22–1.10)
Vulgaris	2.42 (1.15–5.12)	2.44 (1.16–5.17)
Segmental	2.57 (0.30–21.94)	2.59 (0.30–22.23)
Eruptive	2.12 (0.24–18.67)	2.19 (0.25–19.39)
Lip-tip	0.50 (0.17–1.42)	0.51 (0.17–1.48)
Universal	0.41 (0.06–2.96)	0.38 (0.05–2.80)
Trichrome		
No	Reference	Reference
Yes	1.63 (0.52–5.21)	1.67 (0.52–5.35)
Depigmented		
No	Reference	Reference
Yes	0.82 (0.42–1.60)	0.85 (0.43–1.68)
Hypopigmented		
No	Reference	Reference
Yes	0.39 (0.09–1.65)	0.38 (0.09–1.61)
Previtiligo		
No	Reference	Reference
Yes	0.41 (0.03–6.69)	0.41 (0.03–6.78)
Leucotrichia		
No	Reference	Reference
Yes	0.50 (0.13–1.94)	0.53 (0.13–2.11)
Follicular repigmentation		
No	Reference	Reference
Yes	1.68 (0.18–15.46)	1.87 (0.20–17.67)
Koebner's phenomenon		
No	Reference	Reference
Yes	1.38 (0.48–4.00)	1.57 (0.52–4.77)

*Adjusted for age and sex.
